# Prevention of Congenital Transmission of Malaria in Sub-Saharan African Countries: Challenges and Implications for Health System Strengthening

**DOI:** 10.1155/2012/648456

**Published:** 2011-09-22

**Authors:** Kayode O. Osungbade, Olubunmi O. Oladunjoye

**Affiliations:** ^1^Department of Health Policy and Management, Faculty of Public Health, College of Medicine and University College Hospital, University of Ibadan, PMB 5017 General Post Office, 200212 Ibadan, Nigeria; ^2^Department of Community Medicine, University College Hospital, PMB 5116, 200212 Ibadan, Nigeria

## Abstract

*Objectives*. Review of burden of congenital transmission of malaria, challenges of preventive measures, and implications for health system strengthening in sub-Saharan Africa. *Methods*. Literature from Pubmed (MEDLINE), Biomed central, Google Scholar, and Cochrane Database were reviewed. *Results*. The prevalence of congenital malaria in sub-Saharan Africa ranges from 0 to 23%. Diagnosis and existing preventive measures are constantly hindered by weak health systems and sociocultural issues. WHO strategic framework for prevention: intermittent preventive therapy (IPT), insecticide-treated nets (ITNs), and case management of malaria illness and anaemia remain highly promising; though, specific interventions are required to strengthen the health systems in order to improve the effectiveness of these measures. *Conclusion*. Congenital malaria remains a public health burden in sub-Saharan Africa. Overcoming the challenges of the preventive measures hinges on the ability of national governments and development partners in responding to the weak health systems.

## 1. Introduction

Malaria remains a significant burden in sub-Saharan Africa as it continues to be the leading cause of infant morbidity and mortality in Africa. It is believed that malaria contributes up to about 25% of infant mortality in Nigeria [1]. Furthermore, in areas of Africa with stable transmission, *Plasmodium falciparum* infection during pregnancy is estimated to cause as many as 10,000 maternal deaths each year, 8% to 14% of all low birth weight babies, and 3% to 8% of all infant deaths [2]. Thus, malaria burden, if not reduced, poses a threat to the attainment of the Millennium Development Goals 4 and 6.

Congenital malaria is defined as malaria in a newborn or infant, transmitted from the mother [3]. Congenital malaria is generally defined as malaria acquired by the fetus or newborn directly from the mother, either *in utero *or during delivery [4]. It is also defined as the presence of malarial parasites in the peripheral smear of the newborn from twenty four hours to seven days of life [5]. Malaria is considered to be congenital in the neonate when asexual parasites are detected in the peripheral blood within the first week of life [6, 7]. 

Congenital malaria was thought to be rare in developing countries [8, 9]. This might be due to a number of reasons. Firstly, the protective effect of the foetal haemoglobin (HbF) in a newborn is expected to exert its influence during the immediate neonatal period [10, 11]. Secondly, local health facilities in resource-limited settings often lack the capacity to diagnose malarial infection [12–14]. Thirdly, the clinical signs of neonatal malaria may be indistinguishable from those of neonatal sepsis [15]. Thus, congenital malaria might pass unnoticed, and this might be responsible for its underreporting. Therefore, the aim of this paper was to highlight the prevalence and burden of congenital malaria in sub-Saharan Africa as well as the challenges of its diagnosis and prevention; the findings were used to suggest measures aimed at strengthening health systems and reducing the burden of congenital malaria in the population.

## 2. Methods

Literature providing information on the prevalence, burden, diagnosis, prevention, and control of congenital malaria, published between 2000 and 2010 was reviewed. Search terms included “congenital,” “Malaria,” “burden,” “diagnosis,” “prevention”, “control,” and “sub-Saharan Africa.” These literatures were accessed from Pubmed (MEDLINE), Biomed central, Google Scholar, and Cochrane Database of Systematic Reviews. Searches were also supplemented with recommendations from outside experts, reviews of bibliographies of other relevant articles, and systematic reviews.

## 3. Results

### 3.1. Prevalence of Congenital Malaria in Sub-Saharan African Countries

Congenital malaria is rare in developed countries such as the United States of America, where only three infants out of 4.1 million live births were reported to have congenital malaria [16]. The situation is however different and widely varied in sub-Saharan African countries. To this end, recent reports have suggested that congenital malaria is not as rare in developing countries as previously thought [17]. For example, in a survey covering seven sites in sub-Saharan Africa, Fischer showed a mean prevalence rate of 7% for congenital malaria (range 0–23%) [9]. 

Furthermore, while a study in Kenya reported a prevalence of malarial parasitaemia in <0.5% of neonatal admissions [18], high prevalence of congenital and neonatal parasitaemia (>20%) was reported in Uganda and Zambia [19]. A study done in Ghana revealed an incidence of 13.6% congenital malaria infections [20]. In Nigeria, congenital malaria was documented in 13.6% of babies at delivery [21]. In another multicentre study in Nigeria, the overall incidence of congenital malaria was 5.1% (range 1.1%–11.5%) [6]. In addition, Runsewe-Abiodun et al. have reported a congenital malaria prevalence rate of 17.4% among sick neonates in a tertiary hospital in southwestern Nigeria [17].

### 3.2. Burden of Congenital Malaria in Sub-Saharan Africa

It is estimated that placental malaria is responsible for 35% of preventable low birth weight in developing countries [22]. It is also associated with intrauterine growth restriction, prematurity due to preterm labour and intrauterine foetal death [23]. Malaria in pregnancy is reported to cause between 75,000 and 200,000 infant deaths each year in sub-Saharan Africa [22].

### 3.3. Challenges in Detection of Congenital Malaria

The detection of malaria parasites in the infant's blood is essential for diagnosis, although blood smears can be negative if there are low parasite counts (50 parasites/*μ*L blood) [23]. Pengsaa suggested that three repeated blood smears over 48 hours should be reported negative before excluding the diagnosis [19]. The capacity to conduct blood test for the diagnosis of congenital malaria is however limited in sub-Saharan African countries. This is because studies have shown a persistent lack of capacity to conduct quality malaria diagnostic tests by local health facilities [12–14].

Cord malaria can also be detected by polymerase chain reaction (PCR). The use of PCR has suggested that congenital malaria may be more frequent, although it is unclear if a positive PCR represents an active infection [4]. Though, PCR is a more sensitive and accurate diagnostic technique than blood smear microscopy, its use is however very limited in developing countries as it is expensive and requires a specialized laboratory [4]. 

Apart from the laboratory constraints, low antenatal care attendance and skilled delivery rates in African countries will also affect detection and prevention of congenital malaria. Antenatal care utilization in the developing countries is about 65%; this is low compared to that of the developed countries, which is 97% [24]. The implication is that in about two thirds of pregnant women in sub-Saharan Africa, opportunities for early recognition and prompt treatment of malaria infection are missed.

### 3.4. Challenges in the Clinical Diagnosis

The clinical diagnosis of congenital malaria usually poses a challenge. This is because its clinical findings may be indistinguishable from those of neonatal sepsis [15]; hence, the condition is either detected late or not even suspected unless neonates are specifically screened for it. Furthermore, as with many congenital infections, the new born child can manifest with fever, irritability, feeding problems, hepatosplenomegaly, anemia, jaundice, and low birth weight [25]. 

Thus, the clinical distinction from other congenital infections rests primarily on maternal history of exposure to malaria and absence of a skin rash [23]. A study done in Calabar, Nigeria, reported that 35% of newborns with clinical signs of neonatal sepsis were found to have congenital malaria [26]. Fever was the most common feature reported by studies in Kenya and Nigeria among others, which include refusal to feed or poor feeding, irritability, anaemia, hepatosplenomegaly, and jaundice [6].

### 3.5. Challenges in Prevention of Congenital Malaria in Sub-Saharan African Countries

Prevention of any disease condition requires the availability of methods for prediction of those at high risk of the disorder. If there were tests which are adequately sensitive for detecting placenta malaria in the antenatal period, it would have helped in assessing the efficacy of antimalarial drug during pregnancy and identifying the infants at risk of congenital malaria [27]. However, there are no tests which are adequately sensitive for detecting placental malaria; hence, the difficulty in identifying those at high risk of this condition. 

Therefore, malaria preventive measures in pregnancy still remain as priority interventions required to protect the foetus and the newborn against the adverse effects of congenital malaria. The World Health Organization (WHO) has recommended a three-pronged strategic framework in areas of high or moderate (stable) malaria transmission of sub-Saharan Africa: intermittent preventive therapy (IPT), insecticide-treated nets (ITNs), and case management of malaria illness and anaemia [28]. 

#### 3.5.1. Intermittent Preventive Therapy (IPT)

Intermittent preventive therapy (IPT), also known as chemoprophylaxis for pregnant women, especially those in their first pregnancies, has been widely used in sub-Saharan Africa. In line with WHO recommendation, most national guidelines stipulate that all pregnant women should receive at least two doses of IPT given as sulphadoxine-pyrimethamine (SP) combination after quickening as part of preventive treatments at antenatal care [28]. Evidence abound that this measure has recorded successes. For example, researchers in Kenya and Malawi have shown that intermittent preventive therapy with sulphadoxine-pyrimethamine significantly reduces the prevalence of maternal anaemia and placental parasitaemia, and the incidence of low birth weight [29–32]. Furthermore, sulphadoxine-pyrimethamine has been found to be safe in pregnancy and efficacious in reproductive-age women; hence, its strong acceptance and coverage levels of greater than 80% for the first dose [29].

Despite the beneficial impact of sulphadoxine-pyrimethamine on maternal and infant health, its utilization is threatened by weak health systems and sociocultural issues in sub-Saharan Africa. Studies have shown that a substantial proportion (20% to 80%) of pregnant women in this setting make their first antenatal visit in their third trimester [33–35]. Furthermore, unbooked pregnancies are common [36, 37], and antenatal care utilization rate (i.e., antenatal care clinic attendance of at least once during most recent pregnancy) varies from 60% to 90% [1, 38, 39]. These findings could be partly attributed to low quality of service and sociocultural reasons. While evidence abound on the persistent declining quality of antenatal services provided to pregnant women [33, 35, 40], other researchers have found that perceived quality of service was the most important factor which influenced the choice of facility for obstetric care [41]; similarly, a perceived lack of quality in the antenatal care was associated with a late first antenatal visit in Kenya [35]. 

Within the sociocultural context, it is thought that noninitiation of care in the first trimester seems to be a widespread cultural practice in sub-Saharan Africa. In rural Gambia, women do not usually “announce” pregnancy but wait for other family members to discover it, thereby presenting for care well into the third trimester [42]. While further qualitative research is required to explore the extent to which late first antenatal visit is affected by cultural practices, the implication of the findings is that a substantial proportion of pregnant women always remains unprotected against malaria infection because of missed opportunity of treatment in the second trimester. This therefore exposes fetuses of such unprotected women to placental malaria and its adverse effects, thereby contributing to the challenges militating against prevention of congenital malaria. This situation is further worsened by the practice of partial preventive treatments, including sulphadoxine-pyrimethamine (SP) therapy provided by health workers at antenatal care service [33, 35, 43].

#### 3.5.2. Insecticide-Treated Nets (ITNs)

Randomized control trials in sub-Saharan Africa have consistently shown the effectiveness of insecticide-treated nets (ITNs) in the prevention of malaria in pregnancy. Studies in Ghana and Kenya have documented reduced placental malaria, low birth weight, and fetal loss resulting from use of ITNs [44]. Though the use of insecticide-treated nets (ITNs) is reportedly considered as the most cost-effective method of malaria prevention in highly endemic areas, there have always been some concerns about the preventive strategy in sub-Saharan Africa; these concerns include access, cost, retreatment, and gaps between ownership and use of ITN. 

Access to ITNs by vulnerable populations including pregnant women continues to increase due to the efforts of national governments and supporting development agencies through multiple approaches such as stand-alone campaigns, health facilities, and antenatal clinic [1, 38]. Access is also enhanced by free distribution and sales of ITNs at subsidized cost or through discounted voucher systems [1, 38, 45], while the introduction of long-lasting insecticidal nets (LLINs) has reduced the burden of frequent retreatments. 

Despite these efforts, recent findings revealed low utilization of ITNs among pregnant women. Though increasing trends in ownership and use of ITNs by households were reported in national demographic surveys, the reports indicated that 5% to 57% of pregnant women aged 15–49 years slept under an ITN the past night. Furthermore, gaps between ownership and use of an ITN continue to exist as about 50% or less of pregnant women aged 15–49 years in households with an ITN slept under the net the past night [1, 38, 46, 47].

#### 3.5.3. Case Management of Malaria Illness

Supporting and promoting access to correct, affordable, and appropriate treatment within 24 hours of the onset of symptoms of malaria is the third essential component of malaria prevention and control during pregnancy in malarious endemic countries of sub-Saharan Africa [28]. The World Health Organization (WHO) had recommended chloroquine (CQ) in chloroquine-sensitive areas and sulphadoxine-pyrimethamine (SP) in areas with CQ resistance for treatment of uncomplicated malaria in pregnancy. Quinine is another alternative in areas where both CQ and SP are not effective [28]. 

However, prompt and effective case management of malaria illness is hinged on early and correct diagnosis of the condition. As noted above, this is seriously hindered by lack of capacity to conduct quality malaria diagnostic tests by local health facilities in sub-Saharan African countries [12–14]. While this situation might have justified the presumptive treatment of fever with antimalarials (i.e., treating all febrile episodes suspected of malaria with a full therapeutic dose of antimalarials), such practice might have led to a reduced susceptibility of parasites to the commonly used antimalarial drugs in these settings.

### 3.6. Recommendations

#### 3.6.1. Health System Strengthening

The World Health Organization (WHO) recently recommended prompt parasitologic confirmation by microscopy or alternatively by rapid diagnostic tests (RDTs) in all patients suspected of malaria before treatment is commenced unless parasitological diagnosis is not accessible [48]. Findings from the previous review revealed that many peripheral health facilities in resource-poor settings of sub-Saharan Africa lacked the capacity to conduct quality parasitological diagnosis of malaria by microscopy; this is because the procedure is labour-intensive requiring trained staff and quality equipment [49, 50]. 

On the other hand, malaria rapid diagnostic testing (RDT) has been found useful as an attractive alternative to routine microscopy with good sensitivity and specificity profiles [48, 51]. In addition, RDTs have the potential to be cost-effective, require no capital investment or electricity, are simple to perform, and are easy to interpret [51, 52]. If appropriately introduced and implemented, it also has been shown to have the potential of improving drug-prescribing behaviour of health workers [53], thereby reducing overtreatment of malaria and the problem of drug resistance. 

From the foregoing, utilization of RDTs in resource-poor settings would seem to be an appropriate technology as it has the potential of improving the quality of malaria diagnosis and treatment services provided to all cases of malaria, including pregnant women and newborns. Therefore, it is suggested that national governments and development partners in sub-Saharan Africa should support widespread use of rapid diagnostic tests (RDTs) for malaria diagnosis. The capacity of local health facilities providing maternal and child health services should be strengthened with the provision of adequate supplies of equipment and consumables required to provide the diagnostic services. In addition, targeted trainings and supportive supervision of local health staff are highly desirable in order to meet up with the challenges of newly-assigned task. 

In order to increase uptake of IPTs and facilitate prompt diagnosis and treatment of malaria in pregnancy, national governments and development partners in sub-Saharan African countries should also consider improving the poor maternal health service indicators—nonbooking, late antenatal visits and low antenatal service rate—as an essential task to be concertedly pursued. To achieve this task, strategies aimed at improving maternal health service should be implemented. For example, proven-specific interventions such as the World Health Organization (WHO) training on Life Saving Skills [54] are suggested for use of maternal health service managers in addressing the weak health systems. Periodic trainings conducted for local health staff can help address and strengthen health systems, particularly if targeted at previously reported areas of deficiency such as declining quality of maternal health service, poor record keeping practices of health workers, and poor utilization of intrapartum monitoring tools such as a partograph [33, 34, 55]. 

In addition, health staffs' familiarization and adherence to WHO guidelines on provision of effective, efficient, safe, and culturally appropriate services to pregnant women and newborn under the Integrated Management of Pregnancy and Childbirth (IMPAC) would assist to ensure best practices [56]. It is expected that these efforts would improve utilization of maternal health services, increase IPT uptake, enhance prompt diagnosis and treatment of malaria in pregnancy, and consequently prevent congenital malaria. 

On a long term basis, strategies which would bring about over 80% antenatal service utilization rate should be pursued vigorously. Therefore, it is suggested that redirecting and repackaging health education and service content of antenatal care service using a social marketing approach acceptable within the sociocultural context would be found useful. Therefore, placing the antenatal care service content and its benefits on public agenda through different media and matrices may be helpful in the study setting as opposed to the current practice of restricting information to women who make antenatal visits. 

Despite male economic dominance and decision making power in developing countries, their involvement in reproductive health issues is reportedly low [57]. However, studies had shown that male involvement would significantly improve indicators of maternal health service utilization, [58] and, therefore, it could also improve uptake of IPTs and case management of malaria in pregnancy. Hence, it is recommended that father's roles in maternal health be defined, packaged, and incorporated in the antenatal care service messages meant for dissemination to the public. 

With regard to closing the gap between ownership and use of an ITN, a carefully designed qualitative research may be useful in eliciting the factors responsible or reasons for not sleeping under an ITN despite its availability in the household. While it is suggested that national governments and development partners should not relent on their efforts in making ITNs universally accessible to pregnant women either free of charge or at a subsidized price, experiences of other researchers on factors which promote or inhibit ITN usage may be found useful in packaging educational messages aimed at promoting its usage. ITN usage promoting factors such as high perception on the seriousness of malaria and its effect on pregnant women and children, high perceived benefit of ITNs in protecting children and pregnant women against malaria, and high awareness of the prevention of malaria as a better and cheaper option compared with treatment should be intensified. Whereas inhibitory factors such as fear of the chemical that is used to treat nets and unsupportive spouses should be demystified [59].

## 4. Conclusion

Evidence abound that congenital malaria constitutes a public health burden in sub-Saharan Africa. However, efforts of the national governments and development partners at instituting the recommended cost-effective interventions are continuously thwarted by challenges brought about by weak health systems and sociocultural factors among others, thereby, militating against the progress towards attainment of Millennium Development Goal (MDG) 6 (Indicator 22, Target 8). Health system strengthening and appropriate public health promoting and educating messages delivered through a social marketing approach may be found useful in putting back on track the race towards 2015 with respect to attainment of MDG 6.
